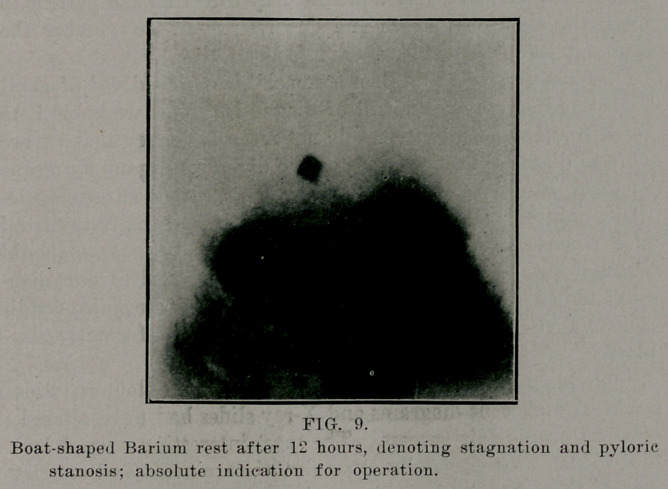# The X-Ray in Diagnosis of Diseases of the Stomach—With Lantern Slide Demonstration

**Published:** 1914-02

**Authors:** L. Amster

**Affiliations:** Diseases of the Digestive Organs and Metabolism, (Piedmont Sanatorium), Atlanta, Georgia


### Journal-Record of Medicine

Successor to Atlanta Medical and Surgical Journal, Established 1855
and Southern Medical Record, Established 1870

**OWNED BY THE ATLANTA MEDICAL JOURNAL COMPANY**

PUBLISHED MONTHLY

*Official Organ Fulton County Medical Society, State Examining Board, Presbyterian Hospital, Atlanta, Birmingham and Atlantic Railroad Surgeons' Association, Chattahoochee Valley Medical and Surgical Association, Etc.*

EDGAR BALLENGER, M. D., Editor
BERNARD WOLFF, M. D., Supervising Editor
A. W. STIRLING, M. D., C. M., D. P. H.; J. S. HURT, B. Ph., M.D.
GEO. M. NILES, M. D., W. J. LOVE, M. D., (Ala.); Associate Editors
E. W. ALLEN, Business Manager

**COLLABORATORS**

DR. W. F. WESTMORLAND, General Surgery
F. W. McRAE, M. D., Abdominal Surgery
H. F. HARRIS, M. D., Pathology and Bacteriology
E. B. BLOCK, M. D., Diseases of the Nervous System
MICHAEL HOKE, M. D., Orthopedic Surgery
CYRUS W. STRICKLER, M. D., Legal Medicine and Medical Legislation
E. C. DAVIS, A. B., M. D., Obstetrics
E. G. JONES, A. B., M. D., Gynecology
R. T. DORSEY, JR., B. S., M. D., Medicine
L. M. GAINES, A. B., M. D., Internal Medicine
GEO. C. MIZELL, M. D., Diseases of the Stomach and Intestines
L. B. CLARKE, M. D., Pediatrics
EDGAR PAULIN, M. D., Opsonic Medicine
THEODORE TOEPEL, M. D., Mechano Therapy
R. R. DALY, M. D., Medical Society
A. W. STIRLING, M. D., Etc., Diseases of the Eye, Ear, Nose and Throat
BERNARD WOLFF, M. D., Diseases of the Skin
E. G. BALLENGER, M. D., Diseases of the Genito-Urinary Organs

VOL. LX ATLANTA, GA., FEBRUARY, 1914. No. 11

### THE X-RAY IN DIAGNOSIS OF DISEASES OF THE STOMACH—WITH LANTERN SLIDE DEMONSTRATION.

DR. L. AMSTER.

DISEASES OF THE DIGESTIVE ORGANS AND METABOLISM, (PIEDMONT SANATORIUM), ATLANTA, GEORGIA.

For a number of years I have been especially interested in Diseases of the Digestive Organs.

In diagnosing my cases I have followed the customary rules of examination: taking the History of my patients, making the physical examination, using, if necessary, the stomach tube, administering different test meals, making chemical and microscopic examination of stomach contents, etc.

Not until the past year, during my stay abroad, did I become interested and grasp the importance of the X-ray for diagnostic purposes.

The more I investigated the field, and after having seen the almost universal adoption, the more convinced I became that the X-ray has taken a permanent place in the diagnosis of Gastro-Enterology. I am indebted for my knowledge of the subject to my chief, Professor Albu, and to Dr. Katz, of Berlin, at whose private Clinic and Polyclinic I had ample opportunity for observation and learning.

Today, I would as little think of getting along without the X-ray apparatus as I would think of dispensing with the microscope in kidney diagnosis.

Fluoroscopy or Radioscopy and Radiography for examining heart and lungs have been in use long before their adoption in gastric work, because there was no means known by which a "Density contrast" could be seen on the Fluoroscopic screen or the X-ray plate. The method of inflation with air, or introducing a stomach tube for the purpose of X-ray examination, did not lead to satisfactory results. Not until the introduction of the so-called "Rieder testmeal" i. e., the addition of a metallic salt of Bismuth to certain foods, was the problem solved of getting a true X-ray picture of Oesophagus, Stomach, and Intestines.

The usual X-ray meal consists of 40 to 50 grams of Bismuth carbonate mixed with some vehicle to make up about 400 grams. The food employed is optional: some use pudding or mush, such as farina, apple sauce, chocolate, etc. A very convenient vehicle is thick fermented milk or Koumyss. In place of Bismuth, many use about 100 grams of Barium Sulphate. This is mostly used in Europe. It meets all requirements and is inexpensive. Substances, such as Zirkonoxide or Contrastin are rarely used.

No matter what one uses, it should be of the same temperature, quantity an consistency, for all examinations, so that by comparison the results may be uniform.

The X-ray examination of the stomach teaches us that the normal active stomach is not located horizontally and shaped like the text books of anatomy describe it and picture it, or as we find it on the post-mortem table. Rieder, of Munich, has demonstrated that in the majority of cases the stomach is a vertical tube and fish-hook shaped, and lies almost entirely on

FIG. 9.

Boat-shaped Barium rest after 12 hours, denoting stagnation and pyloric stanosis; absolute indication for operation.

the left side. According to Holzknecht, of Vienna, the stomach is "steerhorn" shaped. Most observers agree with Rieder, but there are other normal transitional varieties of both shapes, depending on the extent of the filling of the stomach and the shape of the thorax and abdomen.

For the examination of the stomach, Fluoroscopy is most often used. Radiography, or the taking of an X-ray, is used to study the details of the case at one's leisure, and to observe points which may have escaped one's notice at the Fluoroscopic examination. Kinematography is also used, but this procedure is too expensive for ordinary use.

The stomach is always examined in the erect position.

The X-ray, furthermore, enlightens us as to the motility of the stomach, the time it takes food to leave the stomach, the peristaltic action, and the "perystole," i. e., the tonus or the capacity of the musculature to grasp and encircle the food; to see if any pathological condition exists; any tumors within or around the stomach; any adhesions, by directing the patient to draw the stomach upward and downward—to see whether the organ is freely movable.

Formerly the "hourglass stomach" was considered of great rarity and extremely difficult of diagnosis. Now we know it to be of common occurrence. I do not mean to convey that we are able to diagnose every disease of the digestive organs by means of the X-ray alone, neither do I agree with some surgeons who claim that the history of a case, palpation, and the X-ray is all that is necessar y for diagnostic purposes. I believe we should employ every other method available, and confirm our diagnosis by the X-ray. Sometimes we are able to recognize conditions which cannot be recognized by methods formerly employed.

Owing to the different construction of the lantern slide machine, numerous diagrams and X-ray slides had to be altered, some with only fair success. The most interesting cases I had to dispense with especially examples of carcinoma and restrict myself to a few typical illustrations. The cuts in my next paper on Stomach and Intestines will be prepared from original X-ray plates made from my Atlanta material.

215-217 Equitable Building.

## Figures and Tables

**FIG. 1. f1:**
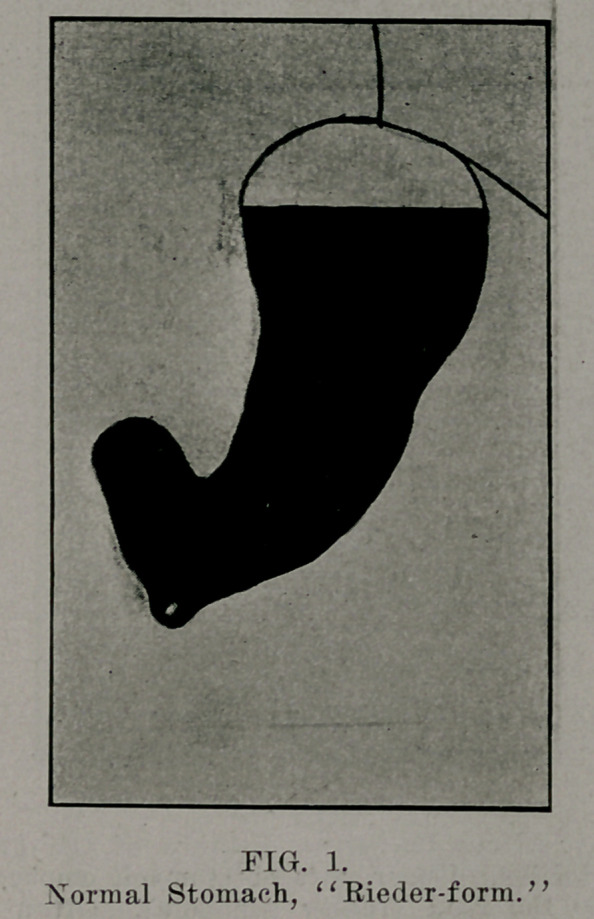


**FIG. 2. f2:**
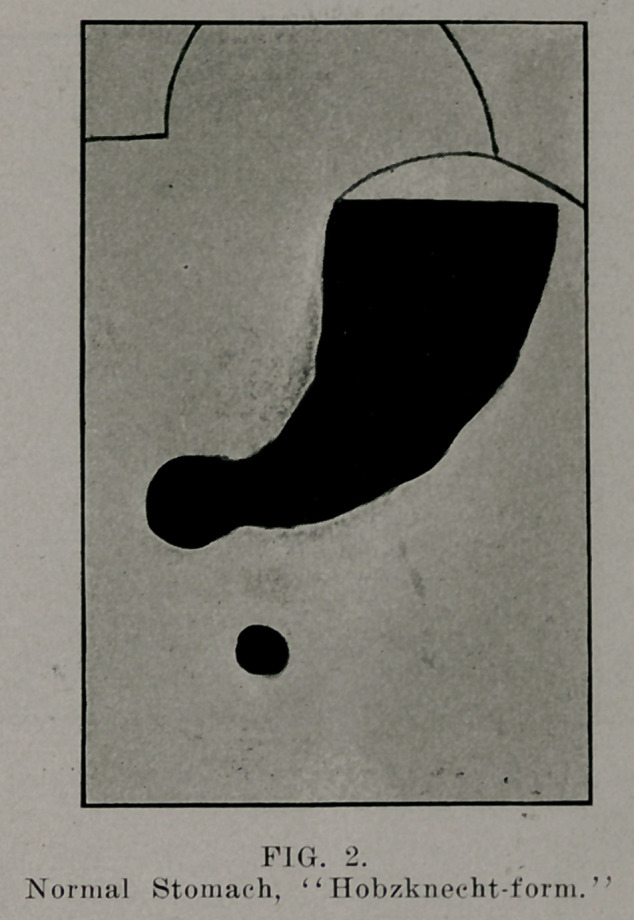


**FIG. 3. f3:**
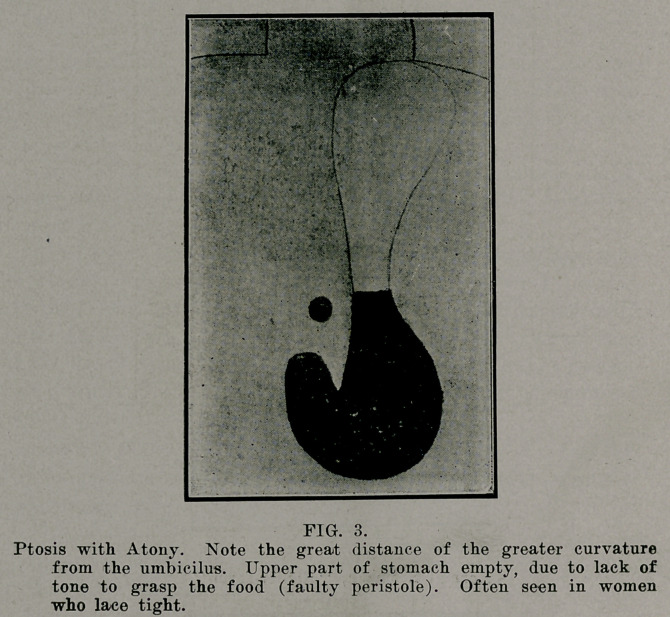


**FIG. 4. f4:**
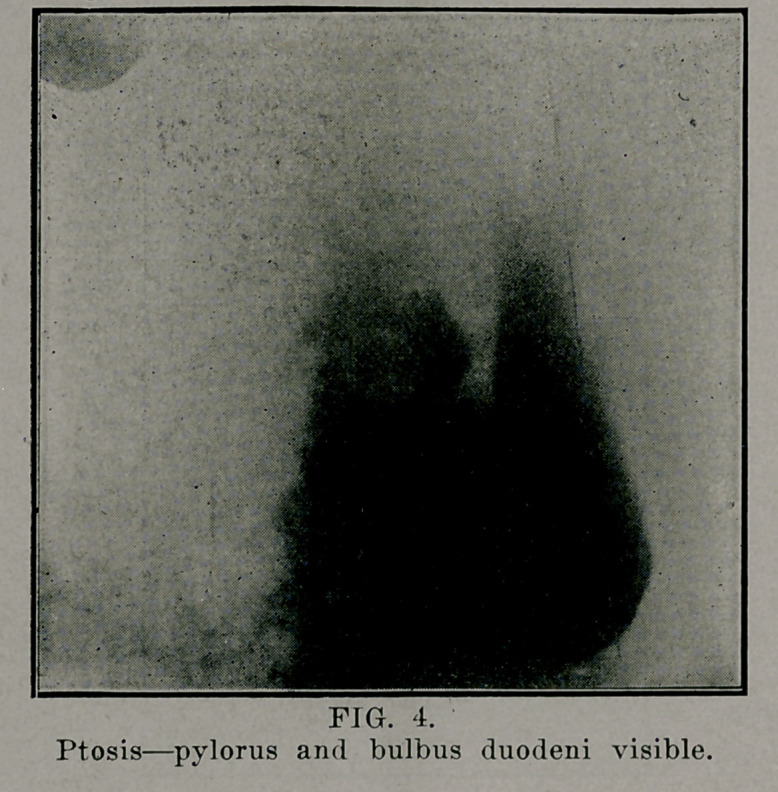


**FIG. 5. f5:**
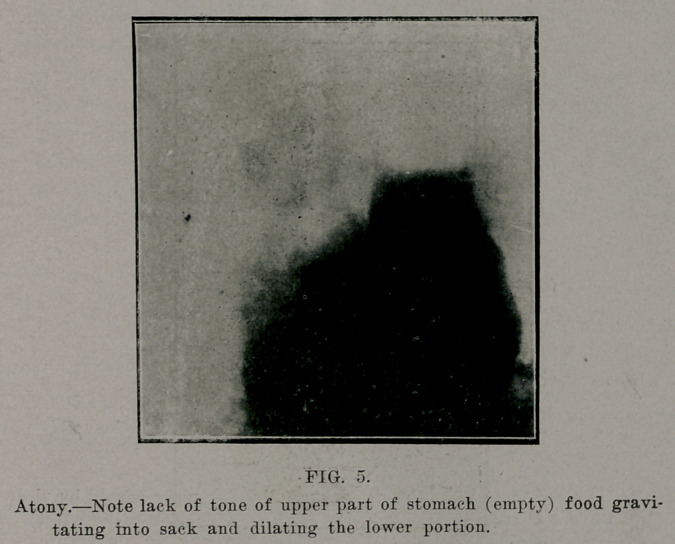


**FIG. 6. f6:**
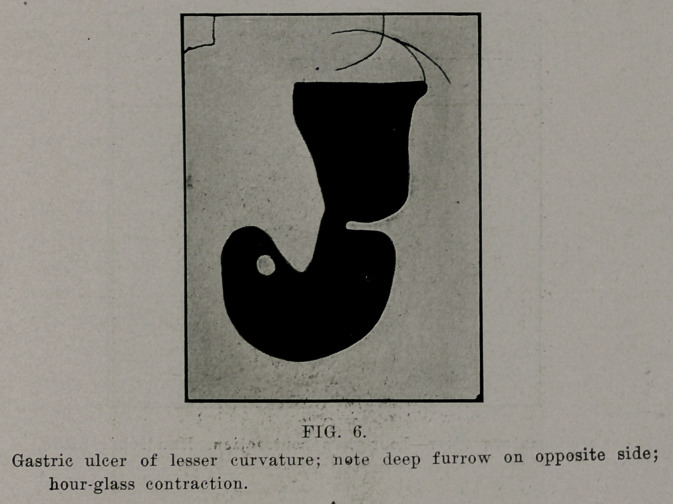


**FIG. 7. f7:**
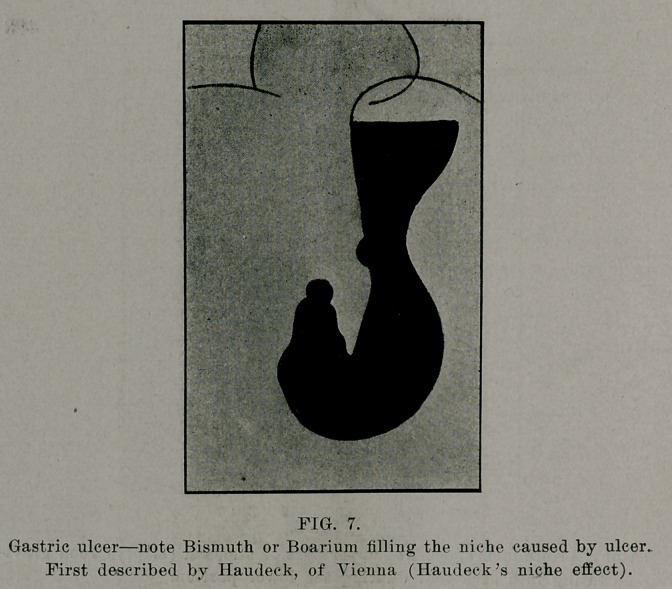


**FIG. 8. f8:**
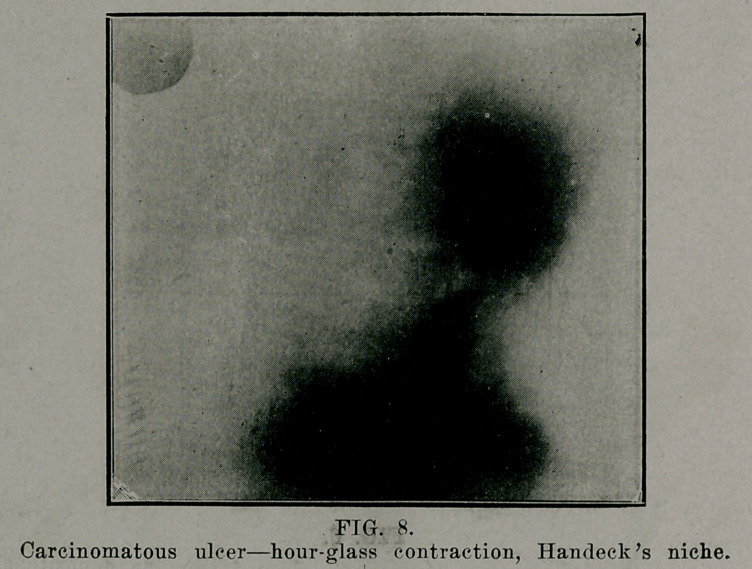


**FIG. 9. f9:**